# Both caffeine and *Capsicum annuum* fruit powder lower blood glucose levels and increase brown adipose tissue temperature in healthy adult males

**DOI:** 10.3389/fphys.2022.870154

**Published:** 2022-08-09

**Authors:** Lachlan Van Schaik, Christine Kettle, Rod Green, Daniel Wundersitz, Brett Gordon, Helen R. Irving, Joseph A. Rathner

**Affiliations:** ^1^ Department of Rural Clinical Sciences, La Trobe Institute for Molecular Science, La Trobe University, Bendigo, VIC, Australia; ^2^ Department of Rural Allied Health, Holsworth Research Initiative, La Trobe Rural Health School, La Trobe University, Bendigo, VIC, Australia; ^3^ Department of Anatomy and Physiology, School of Biomedical Sciences, The University of Melbourne, Melbourne, VIC, Australia

**Keywords:** thermogenesis, substrate utilisation, capsaicin, glucose use, infra-red thermography (IRT), energy expenditure (EE), randomized double-blind placebo and positive-controlled crossover trial

## Abstract

Using a combination of respiratory gas exchange, infrared thermography, and blood glucose (BGL) analysis, we have investigated the impact of *Capsicum annuum (C. annuum)* fruit powder (475 mg) or caffeine (100 mg) on metabolic activity in a placebo controlled (lactose, 100 mg) double-blinded three-way cross-over-design experiment. Metabolic measurements were made on day 1 and day 7 of supplementation in eight adult male participants (22.2 ± 2 years of age, BMI 23 ± 2 kg/m^2^, x̅ ± SD). Participants arrived fasted overnight and were fed a high carbohydrate meal (90 g glucose), raising BGL from fasting baseline (4.4 ± 0.3 mmol/L) to peak BGL (8.5 ± 0.3 mmol/L) 45 min after the meal. Participants consumed the supplement 45 min after the meal, and both caffeine and *C. annuum* fruit powder restored BGL (F _(8,178)_ = 2.2, *p* = 0.02) to near fasting levels within 15 min of supplementation compared to placebo (120 min). In parallel both supplements increased energy expenditure (F _(2, 21)_ = 175.6, *p* < 0.001) over the 120-min test period (caffeine = 50.74 ± 2 kcal/kg/min, *C. annuum* fruit = 50.95 ± 1 kcal/kg/min, placebo = 29.34 ± 1 kcal/kg/min). Both caffeine and *C. annuum* fruit powder increased supraclavicular fossa temperature (F _(2,42)_ = 32, *p* < 0.001) on both day 1 and day 7 of testing over the 120-min test period. No statistical difference in core temperature or reference point temperature, mean arterial pressure or heart rate was observed due to supplementation nor was any statistical difference seen between day 1 and day 7 of intervention. This is important for implementing dietary ingredients as potential metabolism increasing supplements. Together the results imply that through dietary supplements such as caffeine and *C. annuum*, mechanisms for increasing metabolism can be potentially targeted to improve metabolic homeostasis in people.

## Introduction

Brown adipose tissue (BAT) is activated rapidly in response to cold exposure, and diet, coupled with the potential to improve metabolic homeostasis in people with metabolic dysfunction (Poher et al., 2015; Van Schaik et al., 2021a). BAT is a specialised tissue that consumes energy, turning it into heat through non-shivering thermogenesis (Cannon and Nedergaard, 2004). BAT is a highly metabolically active tissue that participates in glucose homeostasis (Matsushita et al., 2014) and removes triglycerides from the blood (Bartelt et al., 2011; Berbee et al., 2015; Khedoe et al., 2015). In humans, as amounts of BAT decrease with age (Yoneshiro et al., 2011), changing the rate of BAT activity, or inducing BAT recruitment may provide considerable benefits for people with metabolic dysfunction. As such, BAT has the potential to not only be therapeutic but also a preventive mediator in lifestyle-related diseases, particularly diabetes mellitus, due to BAT activation improving whole-body glucose homeostasis and insulin sensitivity in humans (Chondronikola et al., 2014). Previous studies have shown cold acclimation increases BAT mass and BAT thermogenesis by modulating and improving insulin sensitivity in healthy humans (1 month acclimation at 19°C) (Lee et al., 2014) or patients with type 2 diabetes mellitus (10 days at 14*–*15°C) (Hanssen et al., 2015). These effects could possibly be achieved through dietary ingredients, as it has been shown that diet can stimulate BAT activity (Hibi et al., 2016; Saito et al., 2020), but the extent to which individual nutrients can have comparable effects is not well established.

Caffeine is the psycho-stimulant component of coffee, and other beverages (Smith, 2002). Caffeine increases thermogenesis in both rodents, and humans (Velickovic et al., 2019; Van Schaik et al., 2021b). However, the dose of caffeine is important, as large doses (≥410 mg) are known to have significant adverse effects on heart rate, blood pressure and be anxiogenic in humans (Noordzij et al., 2005; Vilarim et al., 2011). Conversely, acute single doses comparable to a standard cup of coffee in humans (∼100 mg), increases interscapular BAT temperature without an adverse cardio dynamic effect in male rats (Van Schaik et al., 2021b). A single ingestion of a caffeine capsule (∼375 mg, > 3 cups of coffee) increases the thermogenic activity of BAT in healthy young men and increases energy expenditure in those with only in those who already have high BAT mass compared to those with low BAT mass (Pérez et al., 2021). Glucose homeostasis is altered by acute caffeine ingestion (5 mg/kg) following 2 weeks of daily caffeine consumption in non-caffeine consuming males (Dekker et al., 2007). However, coffee consumption alone is unlikely to elicit significant weight loss in humans due to caffeine-induced lipolysis and catecholamine responses habituation with regular use (Dekker et al., 2007). Together, this suggests that caffeine may not be an anti-obesity therapeutic, but its long-term effect on BAT activity and glucose homeostasis may have considerable benefits for people with metabolic dysfunction. The effects of extensive caffeine use over several days on human BAT activity has not previously been reported.

Red peppers and *Capsicum annuum* (*C. annuum*) fruit are used as spices throughout the world. The major pungent principle of red pepper and *C. annuum* fruit is capsaicin (Saito and Yoneshiro, 2013), which has been reported to elevate body temperature in humans (Hachiya et al., 2007) and stimulate the secretion of catecholamines in rats (Watanabe et al., 1987). Capsaicin or capsinoids activate BAT thermogenesis in BAT positive humans (individuals with active BAT), as measured by [^18^F] fluorodeoxyglucose positron emission tomography-computed tomography (18F-FDG PET/CT) imaging (Sun et al., 2018). Capsaicin activates transient potential receptor vanilloid 1 (TRPV1), which results in increases in adrenaline secretion (Iwasaki et al., 2008) and energy expenditure (Yoneshiro et al., 2012), while potentiating decreases in body fat in humans (Snitker et al., 2008). In mice, TRPV1 channels attenuate diet induced obesity and insulin resistance (Lee et al., 2015), and TRPV1 activation counters diet induced obesity through BAT activation (Baskaran et al., 2017). Furthermore, dietary capsaicin induces browning of white adipose tissue by activating TRPV1 channels (Baskaran et al., 2016; El Hadi et al., 2019). Prolonged ingestion of capsinoids for 6 weeks increases BAT vascular density and resting energy expenditure in healthy adults (Fuse et al., 2020). Capsaicin and its analog capsinoids, are representative TRPV1 agonists, and as such decrease body fat through the activation and recruitment of BAT, mimicking the effects of cold induced thermogenesis (Saito, 2015). While capsinoids/capsaicin activate BAT, the 18F-FDG PET/CT technique does not quantify the extent of thermogenesis or measure acute uptake of free fatty acids as a substrate for heat production. Infra-red thermography (IRT) is an alternative non-invasive imaging technique (Law et al., 2018; Brasil et al., 2020), employing the heat emitting properties of BAT and the superficial position of the supraclavicular human BAT depot. Several research groups have utilised IRT to show a specific rise in temperature in the supraclavicular fossa (Tscf), after cold stimulus and caffeine treatment (Lee et al., 2011; Symonds et al., 2012; Salem et al., 2016; Velickovic et al., 2019; Pérez et al., 2021).

We have shown in rodents that acute central and systemic administration of stimulatory, but non-anxiogenic doses of caffeine activates key hypothalamic nuclei involved in the regulation of BAT and increases interscapular BAT temperature (Van Schaik et al., 2021b). Although acute consumption of coffee increases supraclavicular temperatures in adult humans (Velickovic et al., 2019; Pérez et al., 2021), there is a gap in the understanding of the effect of longer periods of caffeine ingestion (>1 day) on BAT thermogenesis, energy expenditure, and plasma glucose levels. Both caffeine and *C. annuum* in isolation have previously been shown to activate BAT (Yoneshiro et al., 2012; Velickovic et al., 2019), increase energy expenditure (Yoneshiro et al., 2012; Pérez et al., 2021) and improve glucose handling (Dekker et al., 2007; Chaiyasit et al., 2009), but not all in one study. Therefore, *C. annuum* will be used as a positive control. The primary aim of this study is to test whether longer periods of caffeine ingestion activates BAT thermogenesis, increases energy expenditure, lowers respiratory exchange ratio (RER), and lowers plasma glucose levels following a carbohydrate load. A secondary aim of this study is to test the effects of longer periods of caffeine ingestion on sympathetic activity (heart rate and heart rate variability), blood pressure, fat oxidation, and carbohydrate oxidation. Measuring these outcome variables together in the one study is important as it will enable a more holistic physiological assessment of responses to the supplementation. We hypothesise that caffeine ingestion will increase BAT temperature, energy expenditure, and lower blood glucose levels both acutely and on day seven following daily supplementation.

## Research design and methods

The study design and protocol were approved by the University Human Ethics Committee (HEC 19032). Using data published in Acheson (Acheson et al., 2004) the predicated Cohen’s d for difference in carbohydrate metabolism between placebo and caffeine in humans is 0.91. Power analysis using a repeated measure model to determine interaction effect (GPower 3.1.7) predicts that a sample size of eight subjects would allow the study to have 84% power to detect an effect size >0.5 between the supplement and control trials (*p* < 0.05).

### Participants

Eight lean healthy adult male participants aged 18–28 years who could ingest capsules as well as present themselves for six data collection sessions at the University laboratory were recruited to participate in this study (Table 1). Females were excluded from this study as underlying fluctuations due to menstrual cycles prevent accurate evaluation of their energy expenditure changes using the experimental procedures (Sievers et al., 2019). All participants were screened for health status. Exclusion criteria included diabetes mellitus, participants taking any prescription medication, a body mass index (BMI) of >30 kg/m^2^, and an inability to tolerate or experience potential adverse effects from coffee or caffeine containing products.

**TABLE 1:** Participant demographics.

	All participants
*n*	8
Age, *y*	22 ± 2
Height, cm	176 ± 5
Weight, kg	74 ± 8
BMI, kg/m^2^	23 ± 2
Body fat, %	20 ± 8

Values are means ± SD unless otherwise indicated.

### Study design

**FIGURE 1:**
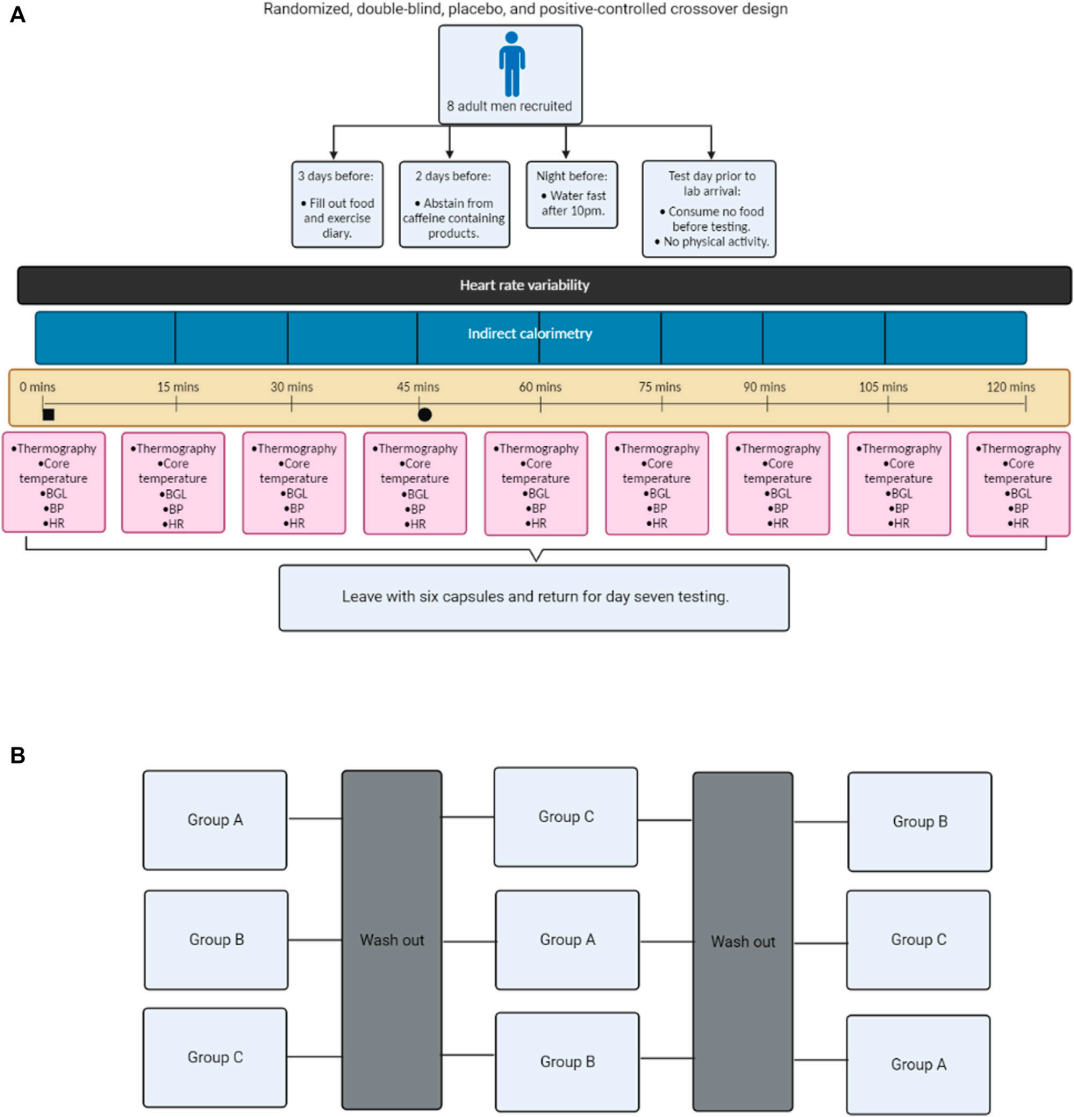
Flow chart schematic of the study design. Experimental process **(A)**; Cross over design **(B)**. Blood glucose levels (BGL), blood pressure (BP), and heart rate (HR). Black square = time of carbohydrate load, Black circle = time of intervention, Group A = Caffeine powder supplementation, Group B = *C. annuum* fruit powder supplementation, and Group C = placebo supplementation. Washout period was a minimum of 7 days.

This randomized, double-blind, placebo, and positive-controlled crossover study was designed to compare the physiological effects of caffeine and *C. annuum* fruit in healthy adult males. The participants were randomly allocated to an intervention sequence via a random number generator by a researcher not involved in data collection. The investigators were blinded to conditions until completion of all data collection and analysis. All experiments were conducted under thermoneutral conditions (22°C) (van Marken Lichtenbelt et al., 2009; Iwen et al., 2017). The participants received either caffeine (100 mg/day, No Doze, Key Pharmaceuticals) (Velickovic et al., 2019; Van Schaik et al., 2021b), placebo (lactose, Ajax Chemicals), or capsicum positive control [475 mg of *C. annuum* fruit powder (capsaicin = 2.375 mg), Nature’s Sunshine] (Lejeune et al., 2003; Johnson, 2007) capsules daily for 7-days each. Each of the interventions are non-prescription supplements and used at the manufacturer’s specifications. To blind the participants and researchers to the supplements being ingested, all supplements were repackaged into generic capsules in advance and the capsules were placed in sealed envelopes by an investigator not involved in the data collection. The experimental protocol consisted of three trials (Figure 1), with each trial lasting for 7 days, with a washout period of 7 days between trials (5 weeks in total). Prior to Trial 1, participants were required to water fast for 11 h prior to testing and not perform any vigorous exercise within 24-h before testing. Additionally, participants were asked to abstain from caffeine and caffeinated products for the duration of the experiment. On Day 1 and Day 7 of each trial participants were asked to attend the laboratory water fasted. Participants arrived at the lab at 8 a.m., to control for daily hormone rhythms. Participants’ height and weight were measured on each day of testing. At each laboratory visit, cardiovascular parameters, IRT, indirect calorimetry, blood glucose, and core temperature were measured every 15-min over a 120-min period. After baseline measurements, participants were carbohydrate loaded through consumption of three carbohydrate gels (90 g glucose, Winners Sports Nutrition). In addition, before or after one of the scheduled testing sessions each participant was required to undergo a dual-energy X-ray absorptiometry (DXA; Hologic Horizon, Hologic Inc., Bedford, MA, United States) scan to measure fat mass. Participants filled out food and exercise diaries during the testing protocol. The participants were instructed to maintain their usual dietary intake, and physical activity during the experimental period. On non-testing days, participants were instructed to consume the supplements in the morning at 9 a.m.

### Indirect calorimetry

Substrate utilization and energy expenditure were estimated from expired gas, measured using a ParvoMedics TrueOne 2400 respiratory gas analyser (ParvoMedics Inc., East Sandy, UT, United States). Upon arrival at the laboratory, participants rested quietly on a plinth in the supine position for at least 30-min. Expired O_2_ and CO_2_ was sampled with 5 s averaging over a 15 min period, energy expenditure, and respiratory exchange ratio were calculated and averaged over the stable final 10 min of this period. Following baseline measurements expired O_2_ and CO_2_ were sampled in 15 min intervals. Carbohydrate and lipid oxidation rates and total energy expenditure were calculated using the non-protein Weir equations (Cunningham, 1990; Peronnet and Massicotte, 1991):

$$\mathrm{Fat}\;\mathrm{oxidation}\;\mathrm{rate}\left(g/\min^{-1}\right)=1.695\;VO_{2}-1.701\;\mathrm{VC}O_{2}$$

$$\mathrm{Carbohydrate}\;\mathrm{oxidation}\;\mathrm{rate}\left(g/\min^{-1}\right)=4.585\;\mathrm{VC}O_{2}-3.226\;VO_{2}$$

$$\mathrm{Energy}\;\mathrm{Expenditure}\;\left(\mathrm{kcal}/\min\right)=3.94\times VO_{2}+1.1\times \mathrm{VC}O_{2}$$

### Core temperature measurements

With participants supine and the head in a neutral position, a non-contact infrared thermometer (Berrcom, JXB-178, Guangdong, China; the stated measurement error of this device is ± 0.2°C) was used to acquire body temperature measurements. Core temperature was not directly measured due to COVID-19 safety restrictions; a non-contact infrared thermometer was used as a measurement of core temperature. The non-contact thermometer was positioned consistently towards the centre of the participant’s forehead.

### Plasma blood glucose measurements

Blood samples were collected *via* finger (capillary) puncture and blood glucose levels were determined using a glucometer (Freestyle Optium Xceed, Abbott Diabetes Care, Alameda, SK, Canada). Blood glucose readings were conducted after expired gas measurements were completed.

### Anthropometric measurements

On each testing day body mass was measured using calibrated scales (Seca 813, Seca, Hamburg, Germany) and followed by measurements of stature using a wall mounted stadiometer (Seca 206, Seca, Hamburg, Germany). Body Mass Index (BMI) was calculated as follows: body mass in kilograms divided by the square of stature in meters (kg/m^2^). Body composition (bone mineral density, fat mass, lean mass, total mass, and total body fat percentage) was measured using DXA analysis (Haarbo et al., 1991).

### Cardiovascular measurements

Systolic and diastolic blood pressure (mmHg) as well as heart rate (beats/min) were measured using an automated sphygmomanometer (Omron SEM-2 advanced, Omron, Kyoto, Japan) after expired gas measurements were completed, and at a similar time (within 3 min) as blood glucose measurements. Heart rate variability (HRV; square milliseconds) was determined from continuous electrocardiogram (ECG) recordings obtained from a five lead ECG (Medilog AR12 plus; Schiller, Germany) as a measure for central autonomic balance. Participants were required to remove clothing above the waist to allow placement of ECG electrodes (Ambu Blue Sensor R, Malaysia). The recorded ECG was analyzed using Medilog Darwin2 software (Professional; Schiller, Germany) and HRV was determined in the spectral domain using Welch’s method, a fast Fourier transform (FFT) width 128 overlap and a 5-min window size. Welch’s method and 128 overlap has been shown to provide a smoothed spectral estimate with clearly outlined peaks in low and high frequency bands (Malik, 1998). The calculation includes low frequency bands (LF), high frequency bands (HF), and the log LF/HF ratio as the log LF/HF ratio conforms the data more readily to a normal distribution.

### Infrared thermography

Every 15-min, participants were asked to sit up in an upright posture looking straight ahead with the chest area to neck region exposed. A thermal imaging camera (FLIR E60, FLIR Systems Australia, Melbourne, Australia) was used to acquire images of the anterior neck and upper chest region. The camera was positioned on a tripod at the level of the neck 1 m from the subject’s face.

### Data/statistical analysis

For analysis of the IRT, bilaterally, three regions of the anterior thorax were chosen for analysis of surface temperature, with the skin overlying BAT in the supraclavicular fossa (SCF) and the lateral region of the neck, with the sternal area considered as a control reference point as this area does not contain BAT (van der Lans et al., 2014; El Hadi et al., 2016). Triangular regions of interest (ROI) were placed in the left and right SCF areas, while a circular ROI was placed over the sternal region. The mean temperature of these ROIs was then extracted for further analysis (van der Lans et al., 2014; El Hadi et al., 2016). The thermal images were analysed to determine mean temperature (°C) using FLIR Research and Development software (FLIR Systems Australia, Melbourne, Australia).

All statistical analysis was conducted using PRISM 9 GraphPad software. Averages from the IRT, core temperature, blood glucose, heart rate, and blood pressure data were taken from the measured single time point. Averages from the RER, fat oxidation, carbohydrate oxidation, and energy expenditure were calculated in 10-min epochs. Averages from HRV were calculated in 5-min epochs. Data are expressed as mean ± SD. To assess if any differences at baseline occurred in each measure in the intervention groups, average baseline IRT temperatures, core temperature and cardiovascular measures prior to supplementation were tested using a one-way ANOVA, Welch’s correction. All averages from outcome measurements are expressed as change from baseline for each intervention group. All data were inspected for normality which indicated parametric distribution, furthermore the central limit theorem states that given sufficient samples, sample distribution will be normal, regardless of the underlying population distribution (Kwak and Kim, 2017). The data values were analysed using repeated measures 3-way analysis of variance (ANOVA; day × treatment × time). Each ANOVA assessed differences between treatments (caffeine, capsaicin, and control), day (1 and 7), and time points. For energy expenditure, we summed the rate of energy expenditure for each group and separated it into pre intervention and post intervention for both day 1 and day 7. Data for each group were analysed using separate two-way repeated measures ANOVA for each testing day (day 1 and day 7). Each ANOVA assessed differences between treatments (caffeine, *C. annuum*, and placebo) and time points (pre vs. post intervention).

To assess if the order of treatment impacted on the study, averages from the IRT, core temperature, blood glucose, heart rate, heart rate variability, blood pressure, RER, fat oxidation, carbohydrate oxidation, and energy expenditure values were analysed in the same manner as the 3-way ANOVA described above. However, rather than assessing difference between treatments, day, and timepoints, each ANOVA assessed differences between trial order, day and timepoints (ANOVA; day × trial × time). If a significant interaction or main effect was found, post hoc analysis was conducted *via* a *t*-test between trials to determine where the significance detected by the ANOVA occurred. For multiple comparisons a Bonferroni correction was applied. Values were considered to indicate statistical significance if *p* < 0.05.

## Results

### Participant’s characteristics

A total of eight participants were included in this study. The participants age, stature, mass, BMI, and body fat percentage can be found in Table 1. Each participant’s baseline temperature, blood glucose levels, and cardiovascular measures were recorded and no significant differences in any blood glucose, cardiovascular or temperature measures were detected (Tables 2, 3; *p* > 0.05, one-way AVOVA).

**TABLE 2:** Average baseline skin and core temperature measures.

	Tscf (°C) day 1	Tscf (°C) day 7	Tcore (°C) day 1	Tcore (°C) day 7	Tref (°C) day 1	Tref (°C) day 7
Caffeine capsule	34.08 ± 0.1	34.03 ± 0.1	36.56 ± 0.3	36.45 ± 0.3	34.16 ± 0.2	34.15 ± 0.1
*C. annuum* fruit capsule	33.98 ± 0.1	33.09 ± 0.1	36.54 ± 0.3	36.40 ± 0.2	34.14 ± 0.1	34.14 ± 0.2
Placebo capsule	33.95 ± 0.1	33.95 ± 0.1	36.45 ± 0.3	36.20 ± 0.3	34.03 ± 0.1	34.08 ± 0.1
*p* value	0.12	0.11	0.76	0.13	0.29	0.57

Average baseline skin temperature measures prior to supplementation. Statistical testing (one-way ANOVA, Welch’s correction) indicates no significant difference (*p* value > 0.05) in base line conditions prior to interventions. Values are means ± SD (*n* = 8 per intervention). Tscf, supraclavicular temperature; Tcore, core temperature; Tref, manubrium temperature.

**TABLE 3:** Average baseline for cardiovascular and blood glucose measures.

	HR (BPM) day 1	HR (BPM) day 7	MAP (mmHg) day 1	MAP (mmHg) day 7	BGL (mmol/L) day 1	BGL (mmol/L) day 7
Caffeine capsule	63.25 ± 10.2	59.87 ± 7.4	85.26 ± 9.8	89.61 ± 9.5	4.25 ± 0.24	4.13 ± 0.32
*C. annuum* fruit capsule	58.12 ± 8.6	58.34 ± 6.9	89.8 ± 8.4	85.42 ± 3.6	4.53 ± 0.16	4.41 ± 0.28
Placebo capsule	66.13 ± 8.4	67.31 ± 8.6	88.53 ± 6.5	90.73 ± 6.1	4.42 ± 0.24	4.38 ± 0.18
*p* value	0.25	0.09	0.92	0.82	0.07	0.22

Average baseline for cardiovascular and blood glucose measures prior to supplementation. Statistical testing indicates no difference in baseline conditions prior to interventions. *p* value is calculated using a one-way ANOVA, Welch’s correction, *n* = 8 per intervention. Values are means ± SD (*n* = 8 per intervention). HR, heart rate (beats per minute); MAP, mean arterial pressure (millimetres of Mercury); BGL, blood glucose levels (millimole per litre).

### Caffeine and *C. annuum* effects on substrate utilization and blood glucose levels

A randomised crossover design administering caffeine, *C. annuum* fruit powder, or placebo interventions to healthy male participants were used to assess any potential differences between acute effects and prolonged daily administration of the interventions after a carbohydrate load on respiratory exchange ratio (RER), substrate utilisation, and blood glucose levels (Figure 1). As this is a crossover study it is important to test if the order of treatment has any influence on the outcomes reported. No effect on the order of treatment was observed in this study (Supplementary Figures S1–S3).

**FIGURE 2:**
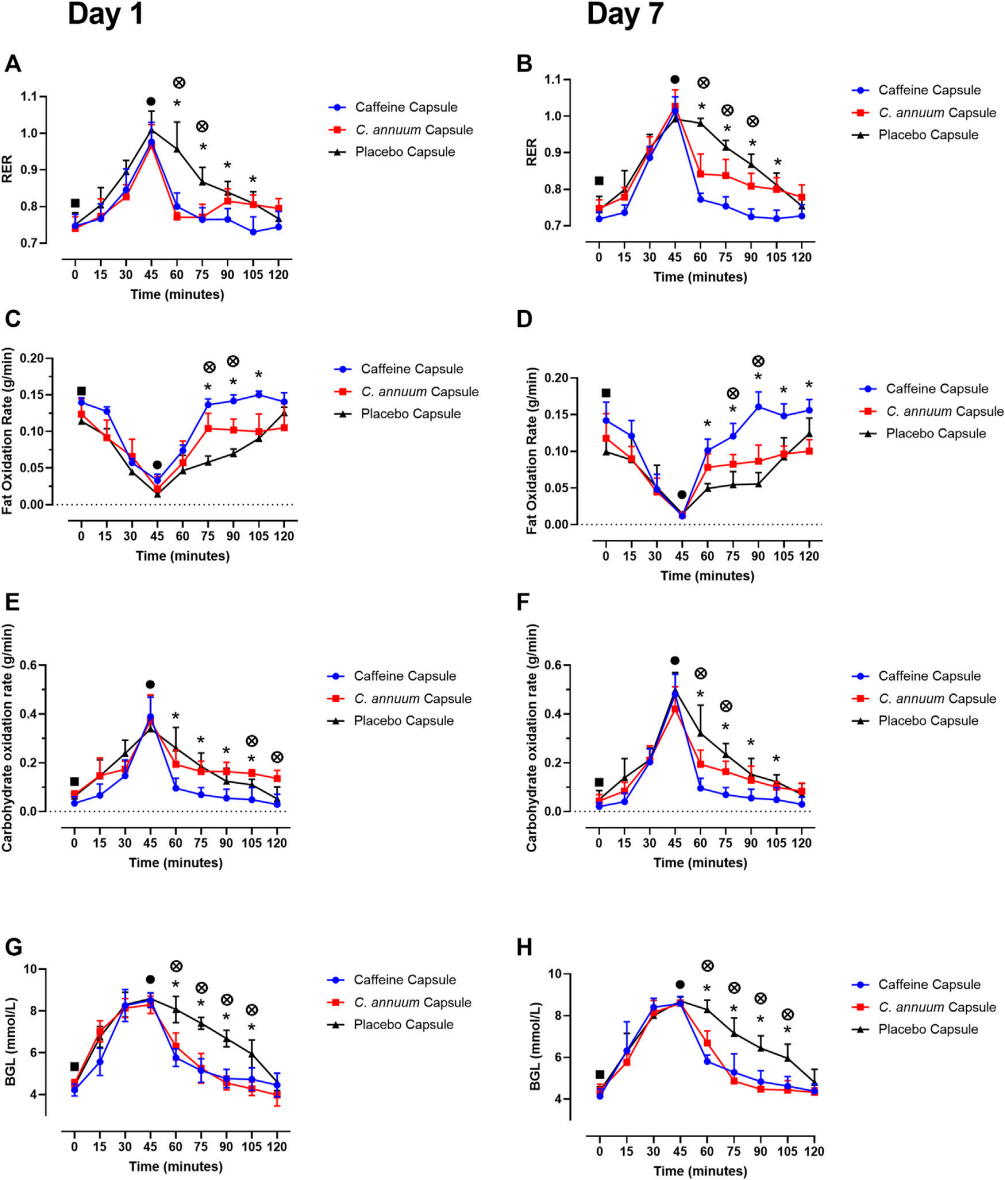
Effects of interventions on metabolic measures. Respiratory exchange ratio (RER) **(A)** day 1 and **(B)** day 7. Fat oxidation rate **(C)** day 1 and **(D)** day 7, and carbohydrate oxidation rate **(E)** day 1, **(F)** day 7. Blood glucose levels **(G)** day 1, and **(H)** day 7. Filled black square, time of carbohydrate load; filled black circle, time of intervention; blue circle, caffeine capsule; red square, *C. annuum* fruit powder capsule; black triangle, placebo capsule. Data is expressed as mean ± SD, *n* = 8 per intervention, *represents caffeine interaction effect, ⊗ represents *C. annuum* fruit powder interaction effects (*, ⊗ *p* < 0.05). Data values were analysed using repeated measures 3-way analysis of variance (ANOVA; day × treatment × time). Each ANOVA assessed differences between treatments (caffeine, *C. annuum* fruit powder, control), day (1 and 7), and time points. If a significant interaction or main effect was found, post hoc analysis was conducted *via* a *t*-test between trials. For multiple comparisons a Bonferroni correction was applied.

For RER a significant interaction effect (F _(9,198)_ = 2.8, *p* < 0.001, Figures 2A,B) was identified. Participants were given a carbohydrate load and then an intervention 45 min later. The caffeine intervention reduced RER from 60 to 105 min, while the *C. annuum* fruit powder intervention lowered RER from 60 to 75 min compared to the placebo at day 1 (Figure 2A). Similar results were seen after 7 days of intervention with the caffeine intervention reducing RER from 60 to 105 min, while *C. annuum* fruit powder improving in efficacy and lowering RER from 60 to 90 min. Reflecting the significant day interactions (F _(9.4,198.5)_ = 2.8, *p* = 0.003, Figures 2A,B). The peak day 1 lowering of RER for the caffeine treatment was 60 min after administration (Figure 2A), peak day 7 lowering of RER for the caffeine treatment was 30 min after administration (Figure 2B). The peak day 1 lowering of RER for the *C. annuum* fruit powder treatment was 30 min after administration (Figure 2A). Peak day 7 lowering of RER for *C. annuum* fruit powder treatment was 75 min after administration (Figure 2B).

For fat oxidation a significant interaction effect (F _(10,229)_ = 8.9, *p* < 0.001, Figures 2C,D) was found. The caffeine intervention increased levels of fat oxidation from 75 to 105 min, while the *C. annuum* fruit powder intervention increased fat oxidation from 75 to 90 min compared to placebo. After 7 days of intervention with caffeine levels of fat oxidation improved with increases between 60 and 120 min, while *C. annuum* fruit powder increased levels of fat oxidation between 75 min and 90 min compared to placebo (Figures 2C,D). The peak day 1 increase in fat oxidation for the caffeine treatment was 60 min after administration (Figure 2C), peak day 7 increase in fat oxidation for the caffeine treatment was 45 min after administration (Figure 2D). The peak day 1 increase in fat oxidation for the *C. annuum* fruit powder treatment was 75 min after administration (Figure 2C). Peak day 7 increase in fat oxidation for *C. annuum* fruit powder treatment was 60 min after administration (Figure 2D). No significant day interaction effect was found for fat oxidation.

For carbohydrate oxidation a significant interaction effect (F _(8,174)_ = 7.1, *p* < 0.001, Figures 2E,F) was found. The caffeine intervention lowered the rate of carbohydrate oxidation between 60 and 105 min, while the *C. annuum* fruit powder intervention lowered the rate of carbohydrate oxidation between 105 and 120 min compared to placebo on day 1 (Figure 2E). Similar results were seen after 7 days of intervention with the caffeine intervention lowering the rate of carbohydrate oxidation between 60 and 105 min, whereas *C. annum* fruit powder lowered the rate of carbohydrate oxidation between 60 and 75 min compared to placebo on day 7 (Figure 2F). The peak day 1 lowering in carbohydrate oxidation for the caffeine treatment was 75 min after administration (Figure 2E), peak day 7 lowering in carbohydrate oxidation for the caffeine treatment was 75 min after administration (Figure 2F). The peak day 1 lowering of carbohydrate oxidation for the *C. annuum* fruit powder treatment was 75 min after administration (Figure 2E). Peak day 7 increase in fat oxidation for *C. annuum* fruit powder treatment was 75 min after administration (Figure 2F). No significant day interaction effect was found for carbohydrate oxidation.

For BGL a significant interaction effect (F _(8,178)_ = 2.2, *p* = 0.02, Figures 2G,H) was found. Participants arrived fasted overnight and were feed a high carbohydrate meal, raising fasting BGL from baseline (4.4 ± 0.3 mmol/L) to peak BGL (8.5 ± 0.3 mmol/L) 45 min after the meal. Participants consumed the supplement at 45 min after the meal, and both caffeine and *C. annuum* fruit powder lowered BGL levels between 60 and 105 min compared to placebo on day 1 and day 7 (Figures 2G,H). Both caffeine and *C. annuum* fruit powder restored BGL to near fasting levels within 15 min of supplementation compared to placebo (120 min) on day 1 and day 7. No significant day interaction effect was found for BGL.

### Caffeine and *C. annuum* effects on total energy expenditure

To assess whether caffeine or *C. annuum* fruit powder increased energy expenditure independent of the carbohydrate load we summed the rate of energy expenditure for each group and separated it into pre intervention and post intervention for both day 1 and day 7.

**FIGURE 3:**
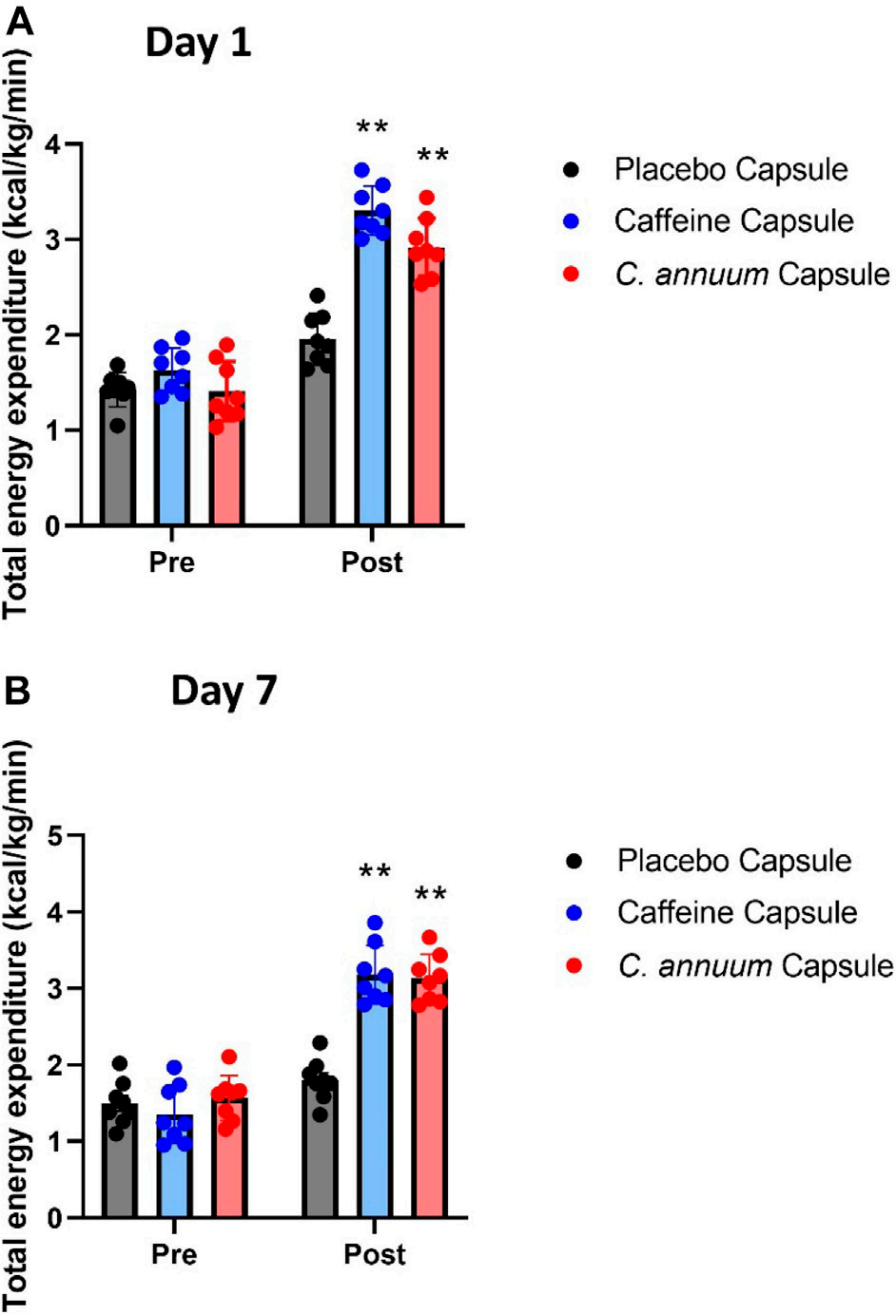
Changes in whole-body energy expenditure in participants between pre- and post-administration of interventions in a 120 min period. **(A)** day 1 and **(B)** day 7. Grey bar, placebo capsule; blue bar, caffeine capsule; red bar, *C. annuum* fruit powder capsule. Data is expressed as mean ± SD, *n* = 8 per intervention, *represents interaction effect (**p < .001). Data values were analysed using repeated measures 2-way analysis of variance (ANOVA; pre × post). Each ANOVA assessed differences between treatments (caffeine, *C. annuum*, and placebo) and time points (pre vs. post intervention).

For day 1 energy expenditure, a significant interaction effect (F_(2, 21)_ = 23.13, p < 0.001, Figure 3A) was found. Post-hoc analysis revealed that both caffeine (3.34 ± 0.13 kcal/kg/min) over the 120-min intervention period and *C. annuum* fruit powder (2.92 ± 0.34 kcal/kg/min over 120 min) significantly increased the rate of energy expenditure compared to placebo (1.95 ± 0.21 kcal/kg/min over 120 min; Figure 3A). Similarly, for day 7, a significant interaction effect (F_(2, 21)_ = 23.13, *p* < 0.001, Figure 3B) was found. Post-hoc analysis revealed that caffeine (3.18 ± 0.16 kcal/kg/min) and *C. annuum* fruit powder (3.13 ± 0.17 kcal/kg/min) significantly increased the rate of energy expenditure compared to placebo (1.8 ± 0.15 kcal/kg/min; Figure 3B). No significant day interaction effect was found for energy expenditure.

### Caffeine and *C. annuum* effects on temperature of the supraclavicular region

**FIGURE 4:**
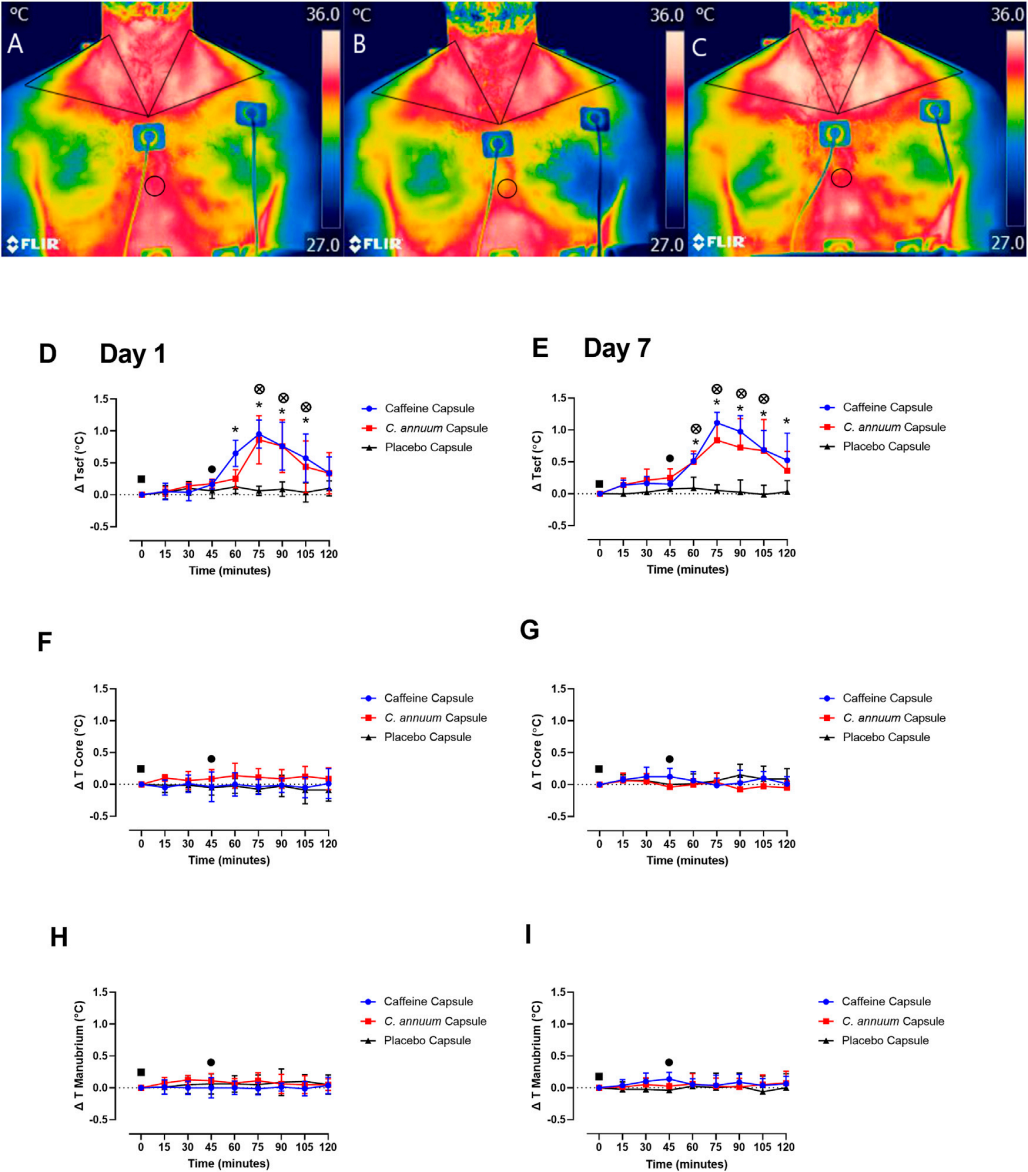
Representative example of thermal images of the skin overlying the region of interest (ROI) located at the supraclavicular fossa (SCF) and sternal (circular ROI) area in participants. **(A)** at baseline measurement, **(B)** 15 min post carbohydrate load and **(C)** at 90 min post intervention with caffeine treatment. Changes in temperature (ΔT) of SCF, core and manubrium in participants following a carbohydrate load (time = 0), and administration of a caffeine capsule, *C. annuum* fruit powder capsule, or placebo capsule (time = 45) to 120 min. SCF temperature **(D)** day 1 and **(E)** day 7. Core temperature **(F)** day 1 and **(G)** day 7, and manubrium temperature **(H)** day 1, **(I)** day 7. Filled black square, time of carbohydrate load; filled black circle, time of intervention; blue circle, caffeine capsule; red square, *C. annuum* fruit powder capsule; black triangle, placebo capsule. Error bars represent S.D., *n* = 8 per intervention, *represents caffeine interaction effect, ^⊗^ represents *C. annuum* fruit powder interaction effects (*, ^⊗^ *p* < 0.05). The data values were analysed using repeated measures 3-way analysis of variance (ANOVA; day × treatment × time). Each ANOVA assessed differences between treatments (caffeine, *C. annuum* fruit powder, control), day (1 and 7), and time points. If a significant interaction or main effect was found, post hoc analysis was conducted *via* a *t*-test between trials. For multiple comparisons a Bonferroni correction was applied. Values were considered to indicate statistical significance if *p* < 0.05.

The supraclavicular region co-locates with BAT in humans (van der Lans et al., 2014; El Hadi et al., 2016), so we utilised IRT to assess whether caffeine or *C. annuum* fruit powder supplementation increased Tscf. Triangular ROIs were placed in the left and right SCF areas, while a circular ROI was placed over the sternal region. IRT images from one participant visually highlights changes in Tscf from baseline (Figure 4A), post carbohydrate load (Figure 4B), and 60 min following caffeine supplementation (Figure 4C). Changes in Tscf following caffeine supplementation are particularly marked (compare Figures 4B,C).

A significant interaction effect (F _(2,42)_ = 32, *p* < 0.001, Figures 4D,E) and post-hoc analysis revealed a single capsule of caffeine or *C. annuum* fruit powder consumed acutely on separate occasions increased Tscf region co-locating with BAT in humans (Figure 4D), and this effect was reproduced at the end of the 7-days treatment period (Figure 4E). Caffeine increased Tscf between 60 and 105 min, while *C. annuum* fruit powder increased Tscf between 75 and 105 min compared to placebo on day 1. For day 7, caffeine increased Tscf between 60 and 120 min, and *C. annuum* fruit powder increased Tscf between 60 and 105 min, with no change in temperature for the placebo treatment. The peak day 1 increase in temperature for the caffeine treatment was 30 min after administration (Figure 4D), similarly peak day 7 increase in temperature for the caffeine treatment was 30 min after administration (Figure 4E). The peak day 1 increase in temperature for the *C. annuum* fruit powder treatment was also 30 min after administration (Figure 4D). Likewise, peak day 7 increase in temperature for *C. annuum* fruit powder treatment was 30 min after administration (Figure 4E). Neither caffeine or *C. annuum* fruit powder treatment evoked changes in core temperature (F _(2,42)_ = 0.5, *p* = 0.5) for both day one and day seven testing (Figures 4F,G). Additionally, there were no changes in temperature of the manubrium (F _(11,240)_ = 1, *p* = 0.2, Figures 4H,I) following treatment of caffeine or *C. annuum* fruit powder on day one and day seven testing. No significant day interaction effect was found for Tscf, core, or manubrium.

### Caffeine or *C. annuum* effects on cardiovascular measures

**FIGURE 5:**
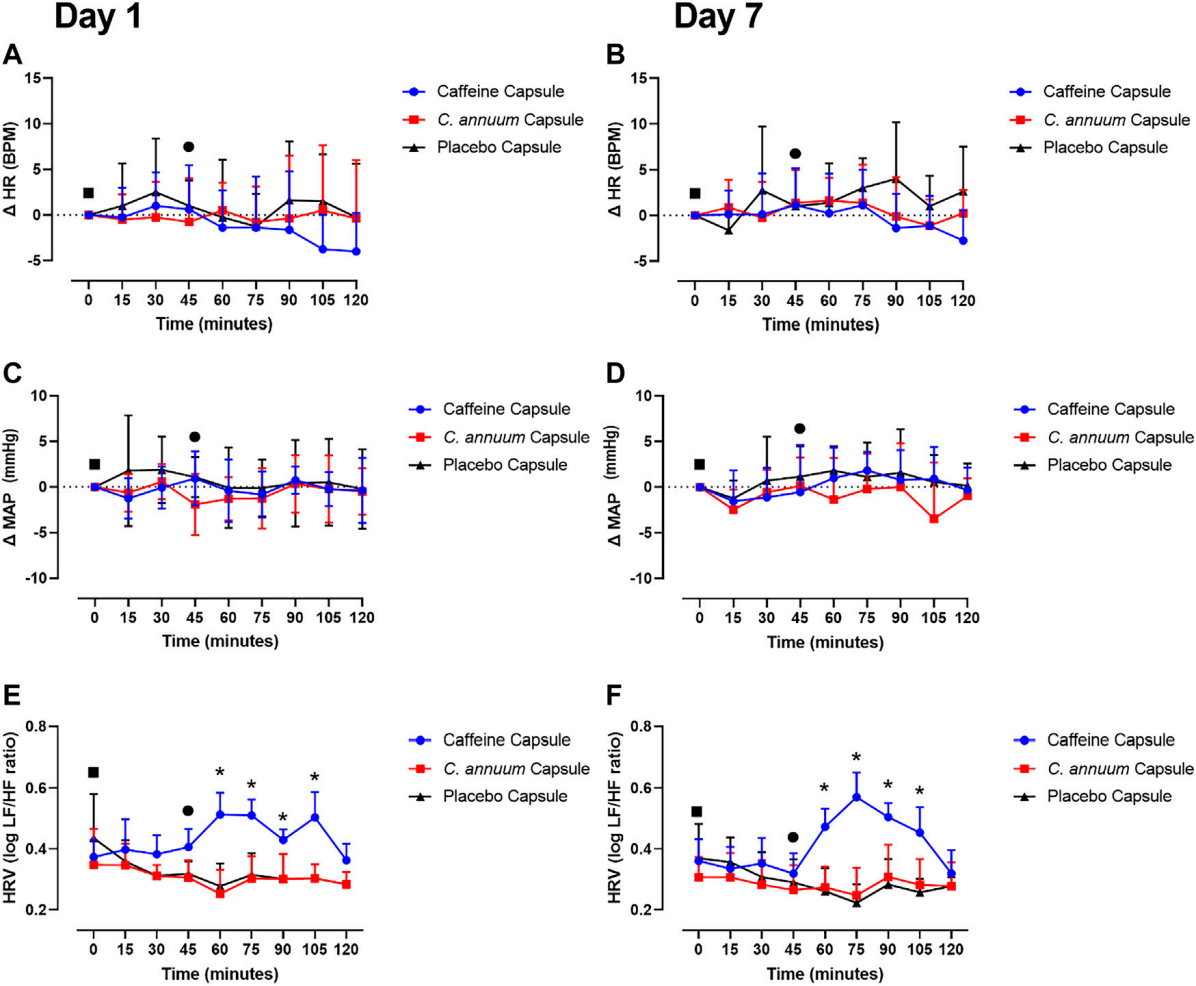
Effects of interventions on cardiovascular measures of participants. The cardiovascular measures were made following a carbohydrate load (time = 0), and administration of a caffeine capsule, *C. annuum* fruit powder capsule, or placebo capsule (time = 45) to 120 min. Changes (Δ) in heart rate **(A)** day 1 and **(B)** day 7. Δ in mean arterial pressure (MAP) **(C)** day 1 and **(D)** day 7, and heart rate variability (HRV) **(E)** day 1, **(F)** day 7. Filled black square = time of carbohydrate load, filled black circle = time of intervention, blue circle = caffeine capsule, red square = capsicum capsule, and black triangle = placebo capsule. Error bars represent S.D., *n* = 8 per intervention, *represents caffeine interaction effect, ⊗ represents capsicum interaction effects (*, ⊗ *p* < 0.05). The data values were analysed using repeated measures 3-way analysis of variance (ANOVA; day × treatment × time). Each ANOVA assessed differences between treatments (caffeine, *C. annuum*, control), day (1, 7), and time points. If a significant interaction or main effect was found, post hoc analysis was conducted using Bonferroni’s test. Values were considered to indicate statistical significance if *p* < 0.05.

To test whether the dose of caffeine and *C. annuum* fruit powder affected cardiovascular responses, heart rate (HR), mean arterial pressure (MAP), and heart rate variability (HRV) were measured. Neither caffeine or *C. annuum* fruit powder changed HR compared to placebo over time on either day 1 or day 7 testing (F _(10,222)_ = 0.6, *p* = 0.7, Figures 5A,B). Similarly, caffeine and *C. annuum* fruit powder had no effect on MAP compared with placebo on both day 1 and day 7 testing (F _(12,268)_ = 0.8, *p* = 0.5, Figures 5C,D). No significant day interaction effect was found for HR and MAP.

For HRV, a significant interaction effect was found (F _(8,170)_ = 1.6, *p* = 0.04, Figures 5E,F). Caffeine treatment significantly increased HRV compared to placebo on day 1 between 60 and 105 min. Similarly, caffeine increased HRV on day 7 between 60 and 105 min, Figures 5E,F). The peak day 1 increase in HRV for the caffeine treatment was 30 min after administration (Figure 5E), peak day 7 increase in HRV for the caffeine treatment was 30 min after administration (Figure 5F). No significant day interaction effect was found for HRV.

## Discussion

This study shows a physiologically significant effect of caffeine and *C. annuum* fruit powder on BAT activation, substrate utilisation, energy expenditure, and blood glucose levels. Here we show that both caffeine (100 mg/day) and *C. annuum* (475 mg/day) taken on separate occasions, lowers blood glucose levels, increases fat oxidation, decreases carbohydrate utilisation, increases total energy expenditure, lowers RER, and increases Tscf after a carbohydrate load. Notably, these effects are sustained in treatments continued over a period of 7 days. To our knowledge this is the first-time measures of energy expenditure, substrate utilisation, blood glucose, and BAT thermogenesis have all together been significantly altered, and this effect has been sustained for a prolonged period.

In this study, we measured thermogenesis as changes in Tscf, a region co-locating with BAT in humans (El Hadi et al., 2016). The increases in Tscf compared to the manubrium reference point and core body temperature, combined with the changes in RER, fat oxidation, and carbohydrate oxidation is indicative of physiologically meaningful BAT thermogenesis following both caffeine and *C. annuum* supplementation. This is important as cold induced BAT thermogenesis improves insulin sensitivity and whole-body glucose homeostasis in healthy individuals (Chondronikola et al., 2014) and those with type 2 diabetes (Hanssen et al., 2015). These results combined with the blood glucose findings suggest that caffeine supplementation through central and peripheral mechanisms (Van Schaik et al., 2021a; Van Schaik et al., 2021b) and *C. annuum* supplementation through TRPV1 mediated mechanisms (Ahuja et al., 2006; Wang et al., 2012; Saito and Yoneshiro, 2013; Baskaran et al., 2016) may have the potential to target BAT to improve insulin sensitivity in humans. The extent to which individual dietary components can activate BAT in humans is not clear. However, the significant results from this study within 15-min following caffeine or *C. annuum* fruit supplementation, may help in providing some clarity about the mechanisms.

Results from this study show that a single caffeine capsule (100 mg) consumed daily increases energy expenditure, Tscf, lowers RER, and increases fat oxidation acutely and after 7 days of supplementation. Pérez et al. (2021) show that in physically active men a single caffeine capsule of 375 mg resulted in an acute increase in energy expenditure 40-min after supplementation and BAT activation (measured via IRT) after 30 min. Results from Pérez et al. (2021) are similar to a previous study (Yoneshiro et al., 2017) which observed an increase in energy expenditure in participants following ingestion of a tea rich in catechins (natural phenolic compounds found in green teas). Our caffeine treatment results are comparable to Pérez et al. (2021) in terms of change in Tscf, and energy expenditure, but importantly we observe this effect for 7 days and at a lower, more tolerable caffeine dose. However, Pérez et al. (2021) assessed the effects of caffeine in physically active men (Pérez et al., 2021). It should be noted that within our study, participants levels of physical activity were collected through food and exercise diaries, and participants were instructed to maintain regular levels of physical activity, but physical activity was not a needed for inclusion in this study. Given this, results from our study significantly build upon those described by Pérez et al. (2021), as a key part of the protocol is the carbohydrate loading of the participants, which ensures carbohydrate metabolism prior to intervention. This was not done in the Pérez et al. (2021) study. BAT thermogenesis switches metabolism from carbohydrate to free fatty acid as evidence by the decline in RER. Although the preferred substrate for BAT thermogenesis is free fatty acids, substantial uptake of glucose into active BAT is well known (Orava et al., 2011; Ouellet et al., 2012; Chen et al., 2013). Thus, we observe concomitant with BAT thermogenesis a fall in blood glucose levels. Both the change in substrate utilization (RER) and the fall in blood glucose levels would not be observable if the in a fasted state.

Human studies investigating the thermogenic influence of nutrients on BAT activity are limited. This may be due to the long considered gold standard to assess BAT function, 18F-FDG PET/CT (van Marken Lichtenbelt et al., 2009) that requires subjects to be fasted. This is due to feeding induced increases in glucose uptake by muscular tissue, which considerably limits the capacity for this method to detect BAT and BAT function (Roman et al., 2015). Additionally, this method alone cannot quantify the physiological significance or amount of BAT activation. Finally, due to the use of ionizing radiation in PET imaging studies, repeat measure cross over designs are difficult to administer. Our technique of carbohydrate loading subjects prior to measuring BAT temperature and combining indirect calorimetry, and blood glucose levels allows us to quantify the physiological extent of thermogenesis and altered substrate utilization, which would otherwise be masked during a fasted state. Several research groups have utilised IRT to show a specific rise in supraclavicular temperatures, after cold stimulus (Lee et al., 2011; Symonds et al., 2012; Andersson et al., 2019). IRT has the advantage of being able to measure BAT activation in real time and can be used repeatedly in large numbers of healthy or unhealthy subjects, irrespective of age or nutritional status. But IRT of SCF alone is not a suitable measure for BAT activation, as it is indirect, and subject to potential confounding factors such as increased blood flow. Temperatures from other landmarks such as the chest region and core temperature are required to demonstrate any increase in Tscf being suggestive of BAT activation (El Hadi et al., 2016; Andersson et al., 2019; Brasil et al., 2020). We have, therefore, utilized a practical method to detect changes in BAT activity, which can detect adjustments in response to ingestion without requiring exposure to radiation. Also, this technique correlates well with the uptake of 18F-FDG following cold exposure (Law et al., 2018).

Our results indicate energy expenditure increases, RER lowers, which coincides with fat oxidation increasing following ingestion of *C. annuum* (capsaicin). A systematic review and meta-analysis (Jang et al., 2020) examined the effects of *C. annuum* supplementation on components of metabolic syndrome. Metabolic syndrome is characterised by hypertension, dyslipidemia, insulin resistance, and abdominal obesity (McCracken et al., 2018). The meta-analysis found *C. annuum* has a significant effect on LDL-cholesterol and body weight, however the meta-analysis did not show any significant effect of *C. annuum* on diastolic blood pressure, and systolic blood pressure, and no significant effect on glucose levels but the analysis showed considerable heterogeneity between studies (Jang et al., 2020). Another meta-analysis which has examined energy expenditure, fat oxidation, and respiratory quotient (Zsiborás et al., 2018) showed that following consumption of capsaicin energy expenditure increased (245 kJ/day, 58.56 kcal/day) and the respiratory quotient decreased (by 0.216) indicating a rise in fat oxidation. Furthermore, these metabolic effects of capsaicin are significant in individuals with a BMI greater than 25 kg/m^2^ (Zsiborás et al., 2018). Although our study only looked at these measures in healthy individuals with a BMI of <25 kg/m^2^, our results are consistent with the findings of this meta-analysis (Figures 2, 3).

Results from this study show that caffeine but not *C. annuum* fruit powder supplement significantly increases HRV (LF/HF ratio) on both testing days. Heart rate variability is the fluctuation in the time intervals between adjacent heartbeats (Ernst, 2017). HRV is a measure of the balance between sympathetic and parasympathetic autonomic drive to the heart (cardiac autonomic activity) (Ernst, 2017). Frequency-domain measurements estimate the distribution of absolute or relative power into four frequency bands. Task Force of the European Society of Cardiology the North American Society of Pacing Electrophysiology (1996) divided heart rate fluctuations into ultra-low-frequency, very-low-frequency, low-frequency (LF), and high-frequency (HF) bands (1996). Essentially, the closer the LF/HF ratio is to one the more sympathetic tone there is, the closer the ratio is to zero the more parasympathetic balance. Results from this study show an increase in HRV (LF/HF ratio) and increased Tscf following caffeine supplementation. This indicates caffeine is acting to increase sympathetic nerve drive systemically in the body (Van Schaik et al., 2021a). Previous rodent studies have shown caffeine works through central mechanisms to activate BAT thermogenesis (Van Schaik et al., 2021b). Notwithstanding the increased sympathetic activity to the heart, the doses of caffeine used in this study had no measurable impact on either HR or MAP. We did not find *C. annuum* to have any effect on HRV (LF/HF ratio). This suggests that the *C. annuum* and caffeine are acting via different pathways to evoke the observed increases in energy expenditure, Tscf and blood glucose removal. We observe that in participants at rest, the LF/HF ratio starts off much lower than we might expect possibly due to participants laying supine rather than sitting upright. This could result in less sympathetic nerve drive required to maintain MAP.

A key strength of this study is that we carbohydrate loaded participants prior to supplementation. Previous studies have used fasted individuals and seen no acute change in substrate utilization, which is masked by the fasted state (Ohnuki et al., 2001; Galgani et al., 2010). By carbohydrate loading the participants we were able to see the oscillation of BGL in response to both carbohydrate loading and supplement intervention along with changes in substrate utilization. Due to COVID-19 safety restrictions core temperature was not measured invasively. As such a non-contact infrared thermometer measuring forehead temperature was used to measure core temperature. These thermometers are calibrated to make clinical measures of fever (Teran et al., 2012). Furthermore, participants in the study laid still at a constant ambient temperature thermoneutral temperature (22°C) (van Marken Lichtenbelt et al., 2009; Iwen et al., 2017), minimising any artefacts due to increased/altered skin blood flow. Evidence suggests that these non-contact thermometers provide good precision in measuring body temperature (Teran et al., 2012; Chen et al., 2020). Although not all studies agree, results from Sullivan et al. (2021) indicate that sensitivity and specificity for predicting a subject’s temperature falls significantly once a 38°C threshold is met. While there may be an offset between core and measured forehead temperature, each of these three papers (Teran et al., 2012; Chen et al., 2020; Sullivan et al., 2021) do indicate that non-contact thermometers are able to measure change in core temperature accurately under the conditions in this study.

Dietary interventions are successful in treating the associated risk factors of metabolic syndrome (Barnard et al., 2009), including the reversal of insulin resistance (Dunaief et al., 2012), increasing insulin sensitivity (Barnard et al., 2005), treating hypertension (Najjar et al., 2018), and reducing body weight (Kahleova et al., 2018). However, individuals may find these types of interventions restrictive and difficult to maintain long term. Consequently, identifying pharmacological therapies that may promote weight loss and treat metabolic disease, through increased energy expenditure *via* thermogenesis, may be a way to augment current interventions for long term weight reduction (Ursino et al., 2009; Nedergaard and Cannon, 2010; Dulloo, 2011; Whittle et al., 2013). Various pharmaceuticals, such as mirabegron and formoterol have been shown to increase energy expenditure, fatty acid oxidation and increase thermogenesis in humans (Lee et al., 2012; O’Mara et al., 2020). But participants report palpitation, tremor, a loss of appetite and insomnia with use of formoterol (Lee et al., 2012), and tachycardia, and raised systolic blood pressure have been observed with use of mirabegron (O'Mara et al., 2020). These side effects will likely reduce the use of these drugs clinically. A possible low cost and low risk option is combining both caffeine and capsaicin. Results from this study indicate that such a combination may be extremely beneficial for individuals with markers of metabolic syndrome. Although we did not measure blood markers for insulin, the rapid return of blood glucose to fasting levels following individual supplementation suggests that it is possible we improved insulin signalling. Additionally, the effects of individual supplementation of caffeine or *C. annum* in relation to RER, energy expenditure and substrate utilisation, and no effect on heart rate and mean arterial pressure show a great benefit with little cardiovascular risk. Interestingly we did find an increase in heart rate variability following caffeine supplementation which adds to an ambiguous body of evidence for caffeine effects on heart rate variability (Rauh et al., 2006; Koenig et al., 2013). A previous study (Yoshioka et al., 2001) examined the combined effects of red pepper and caffeine consumption on 24 h energy balance in subjects given free access to food. Two appetizers (2 × 322 kJ with or without 3 g red pepper) were given before lunch and dinner, and a drink (decaffeinated coffee with or without 200 mg caffeine) was served at all meals and snacks except for the after-dinner snack. An important note is that on the experimental day, 8.6 and 7.2 g red pepper were also added to lunch and dinner respectively. Red pepper and caffeine consumption significantly reduced the cumulative *ad libitum* energy intake and increased energy expenditure. The mean difference in energy balance between both conditions was 4,000 kJ/d (Yoshioka et al., 2001). These results indicate that the consumption of red pepper and caffeine can induce a considerable change in energy balance. Our results are consistent with this observation. There remains potential to replicate the findings from our study, but with the combination of *C. annuum* and caffeine, rather than individual supplementation. Additionally, such a study could be undertaken in healthy subjects or those with metabolic dysfunction.

## Conclusion

In conclusion, our results demonstrate that caffeine and *C. annuum* supplementation increase blood glucose removal, energy expenditure, Tscf, and promote a change in substrate oxidation. These results support a physiological role of caffeine and *C. annuum* on metabolic activity and glucose homeostasis and may provide a basis to pharmacologically target BAT through caffeine and *C. annuum* mediated mechanisms to potentially improve metabolic homeostasis in humans. Further research is needed to investigate the effects of combining caffeine and *C. annuum* on markers of metabolic dysfunction, and the mechanisms underlying BAT activation to identify safe and efficacious lifestyle or pharmaceutical interventions that may activate BAT.

## Data Availability

The raw data supporting the conclusion of this article will be made available by the authors, without undue reservation.
