# Corrigendum: Both caffeine and *Capsicum annuum* fruit powder lower blood glucose levels and increase brown adipose tissue temperature in healthy adult males

**DOI:** 10.3389/fphys.2022.1127730

**Published:** 2023-01-10

**Authors:** Lachlan Van Schaik, Christine Kettle, Rod Green, Daniel Wundersitz, Brett Gordon, Helen R. Irving, Joseph A. Rathner

**Affiliations:** ^1^ Department of Rural Clinical Sciences, La Trobe Institute for Molecular Science, La Trobe University, Bendigo, VIC, Australia; ^2^ Department of Rural Allied Health, Holsworth Research Initiative, La Trobe Rural Health School, La Trobe University, Bendigo, VIC, Australia; ^3^ Department of Anatomy and Physiology, School of Biomedical Sciences, The University of Melbourne, Melbourne, VIC, Australia

**Keywords:** thermogenesis, substrate utilisation, capsaicin, glucose use, infra-red thermography (IRT), energy expenditure (EE), randomized double-blind placebo and positive-controlled crossover trial

In the published article, there was an error in Figure 3 as published. A mistake in the conversion of units due to human error resulted in the values being an order of magnitude too high and the incorrect figure was included in the published article (incorrect and corrected calculation have been provided to the editor/journal for verification). As per the Weir equation (Cunningham, 1990) energy expenditure is calculated in kilocalories (kcal/min). When converting kcal to kilojoules (kJ/min), the correct formula: kcal × 4.184. The authors accidentally used: kJ × 4.184. In an early draft of the manuscript, we converted kcal/kg/min into kJ/kg/min to report the data in SI units, as is required for some publications. In the process a mistake due to human error resulted in the incorrect data being included in Figure 3. The corrected Figure 3 and caption appear below.

**FIGURE 3:**
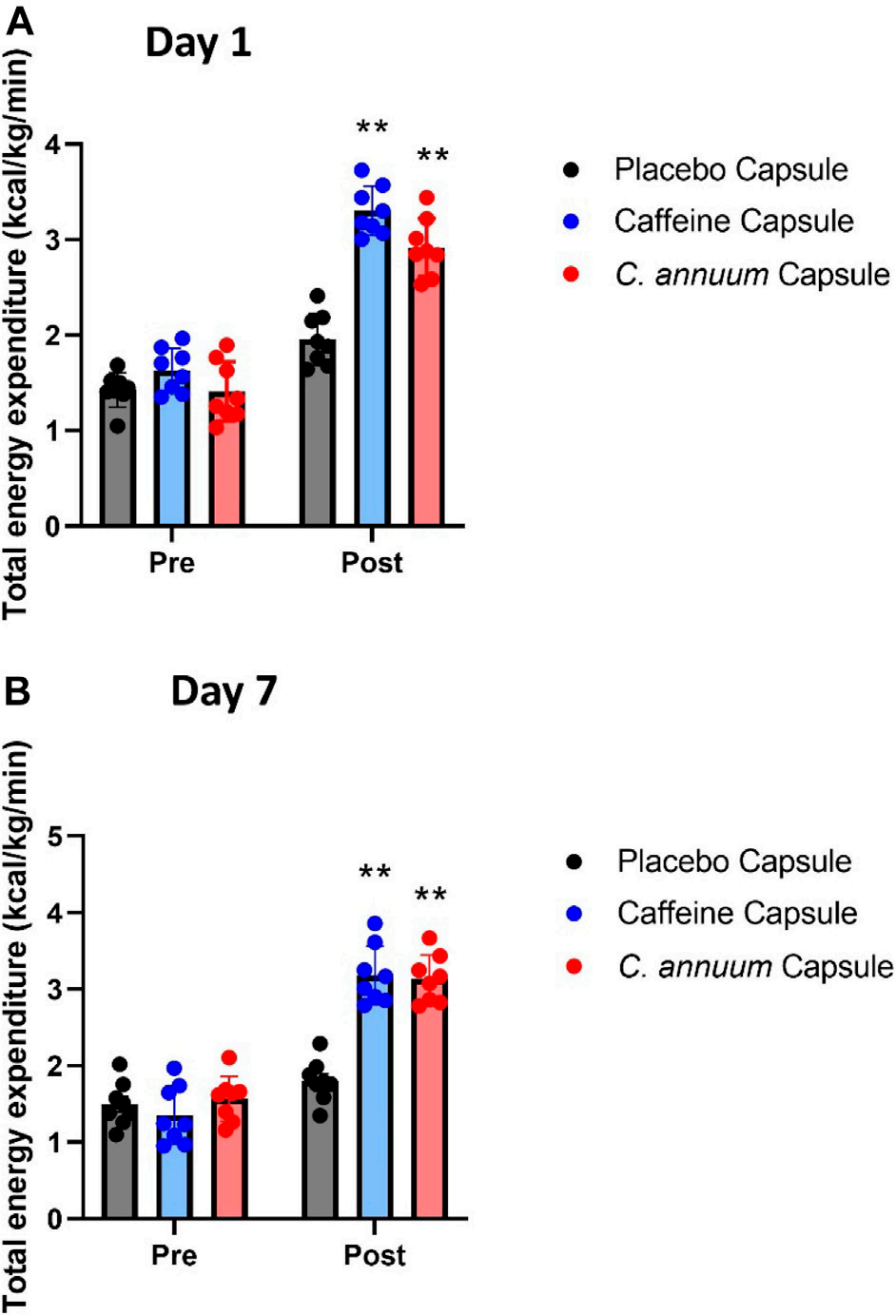
Changes in whole-body energy expenditure in participants between pre- and post-administration of interventions in a 120 min period. **(A)** day 1 and **(B)** day 7. Grey bar, placebo capsule; blue bar, caffeine capsule; red bar, *C. annuum* fruit powder capsule. Data is expressed as mean ± SD, *n* = 8 per intervention, *represents interaction effect (**p < .001). Data values were analysed using repeated measures 2-way analysis of variance (ANOVA; pre × post). Each ANOVA assessed differences between treatments (caffeine, *C. annuum*, and placebo) and time points (pre vs. post intervention).

Also, due to the above error, the results section states incorrect values for the energy expenditure in reference to Figure 3.

A correction has been made to the **results** section, *Caffeine and C. annuum effects on total on total energy expenditure*, paragraph number, 2. The corrected paragraph is given below:

“For day 1 energy expenditure, a significant interaction effect (F _(2, 21)_ = 23.13, *p* < .001, Figure 3A) was found. Post-hoc analysis revealed that both caffeine (3.34 ± .13 kcal/kg/min) over the 120-min intervention period and *C. annuum* fruit powder (2.92 ± .34 kcal/kg/min over 120 min) significantly increased the rate of energy expenditure compared to placebo (1.95 ± .21 kcal/kg/min over 120 min; Figure 3A). Similarly, for day 7, a significant interaction effect (F _(2, 21)_ = 23.13, *p* < .001, Figure 3B) was found. Post-hoc analysis revealed that caffeine (3.18 ± .16 kcal/kg/min) and *C. annuum* fruit powder (3.13 ± .17 kcal/kg/min) significantly increased the rate of energy expenditure compared to placebo (1.8 ± .15 kcal/kg/min; Figure 3B). No significant day interaction effect was found for energy expenditure.”

The authors apologize for this error and state that this does not change the scientific conclusions of the article. The original article has been updated.

