# Supplementary material for: Both caffeine and Capsicum annuum fruit powder lower blood glucose levels and increase brown adipose tissue temperature in healthy adult males
Source: Front Physiol. 2022 Aug 9;13:870154. doi: 10.3389/fphys.2022.870154 (PMC9395699; doi:10.3389/fphys.2022.870154)
Supplement: Supplementary file 1 [file DataSheet1.PDF]

Online Supplemental Materials

# Both caffeine and *capsicum annuum* increase brown adipose tissue temperature and lower blood glucose levels in healthy adult males

Van Schaik L*, Kettle C, Green RA, Wundersitz D, Gordon BA, Irving HR, Rathner JA

Correspondence and requests for materials should be addressed to Mr Lachlan Van Schaik.

(j.vanschaik@latrobe.edu.au)

There are three figures pertaining to trial order data to follow:

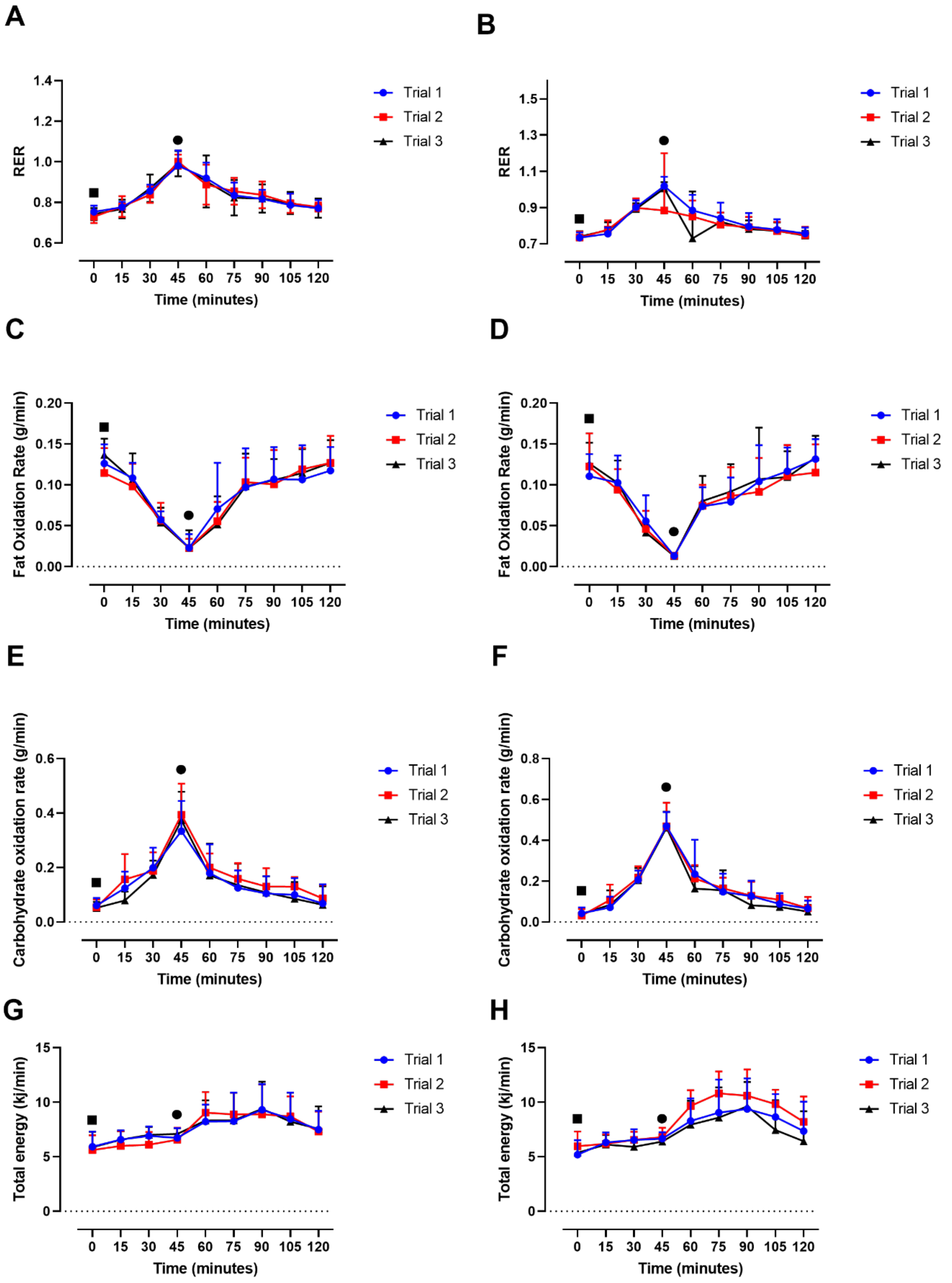

Supplemental Figure 1: Trial order changes in respiratory exchange ratio (RER), fat oxidation rate (g/min), carbohydrate oxidation rate (g/min), total energy (kj/min) in participants following a carbohydrate load (time=0), and administration of a caffeine capsule, capsicum capsule, or placebo capsule (time=45) to 120 min. Respiratory exchange ratio (RER) **(A)** day 1 and **(B)** day 7. Fat oxidation rate **(C)** day 1 and **(D)** day 7, and carbohydrate oxidation rate **(E)** day 1, **(F)** day 7. Energy expenditure

(G) day 1, and **(H)** day 7. Filled black square= time of carbohydrate load, filled black circle= time of intervention, blue circle= trial 1, red square= trial 2, black triangle= trial 3. Error bars represent S.D., n=8 per trial. The data values were analysed using repeated measures 3-way analysis of variance (ANOVA; day x trial x time). Each ANOVA assessed differences between treatments (trial 1, trial 2, and trial 3), day (1, 7), and time points. If a significant interaction or main effect was found, post hoc analysis was conducted via a t-test between trials. For multiple comparisons a Bonferroni correction was applied. Values were considered to indicate statistical significance if $p < 0.05$.

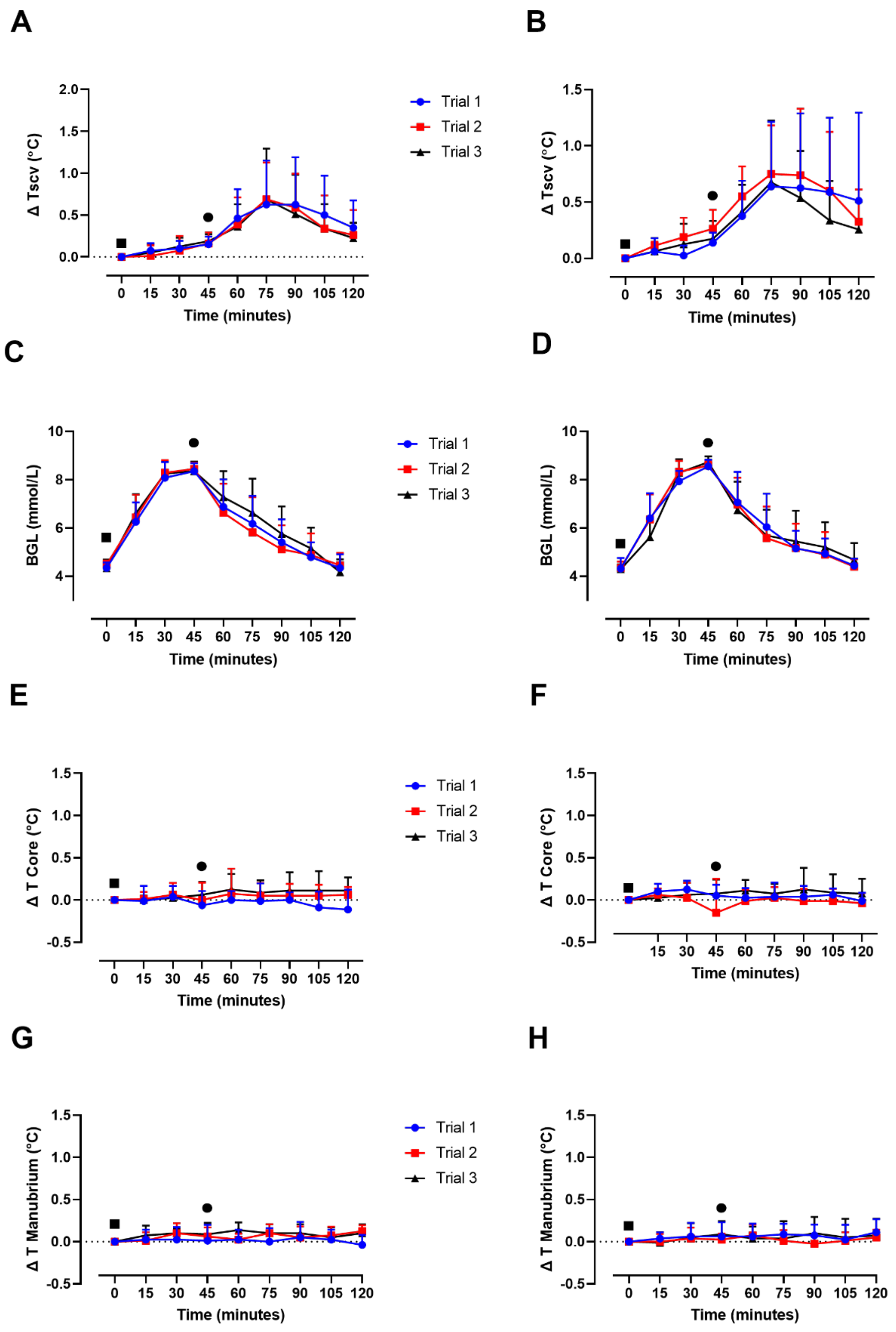
A
B
C
D
E
F
G
H
Δ Tscv (°C)
BGL (mmol/L)
Δ T Core (°C)
Δ T Manubrium (°C)
Time (minutes)
0
15
30
45
60
75
90
105
120
Trial 1
Trial 2
Trial 3

were analysed using repeated measures 3-way analysis of variance (ANOVA; day x trial x time). Each ANOVA assessed differences between treatments (trial 1, trial 2, and trial 3), day (1, 7), and time points. If a significant interaction or main effect was found, post hoc analysis was conducted via a t-test between trials. For multiple comparisons a Bonferroni correction was applied. Values were considered to indicate statistical significance if $p < 0.05$.

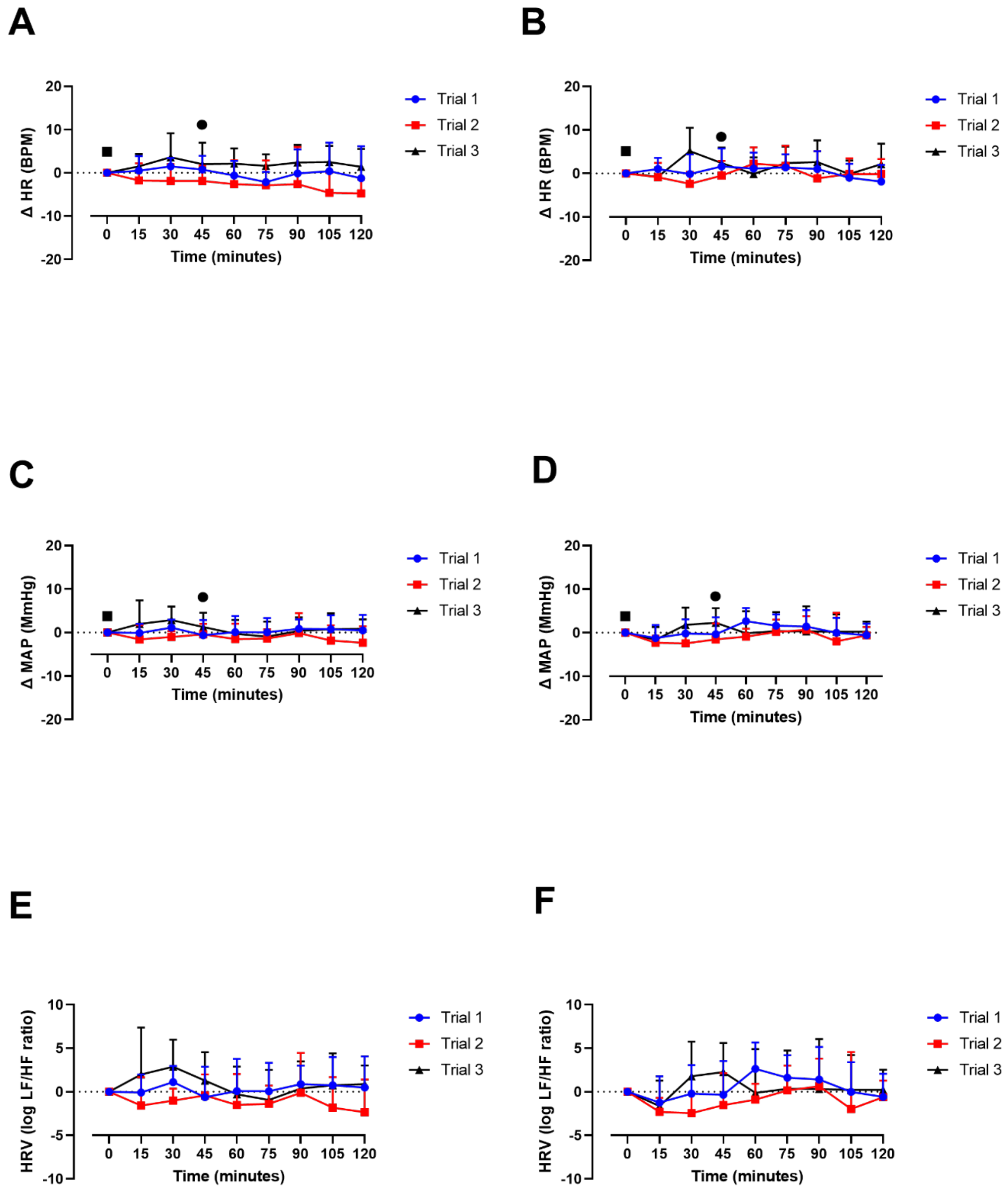

Supplemental Figure 3. Trial order changes in heart rate, mean arterial pressure and heart rate variability in participants following a carbohydrate load (time=0), and administration of a caffeine capsule, capsicum capsule, or placebo capsule (time=45) to 120 min. Δ heart rate **(A)** day 1 and **(B)** day 7. Δ mean arterial pressure **(C)** day 1 and **(D)** day 7, and heart rate variability **(E)** day 1, **(F)** day 7. Filled black square= time of carbohydrate load, filled black circle= time of intervention, blue circle= trial 1, red square= trial 2, black triangle= trial 3. Error bars represent S.D., n=8 per trial. The data values were analysed using repeated measures 3-way analysis of variance (ANOVA; day x trial x time). Each ANOVA assessed differences between treatments (trial 1, trial 2, and trial 3), day (1, 7), and time points. If a significant interaction or main effect was found, post hoc analysis was conducted via a t-test between trials. For multiple comparisons a Bonferroni correction was applied. Values were considered to indicate statistical significance if $p < 0.05$.
